# Supervised Home-Based Exercise Prehabilitation in Unfit Patients Scheduled for Pancreatic Surgery: Protocol for a Multicenter Feasibility Study

**DOI:** 10.2196/46526

**Published:** 2023-09-07

**Authors:** Nicole D Hildebrand, Allard G Wijma, Bart C Bongers, Sander S Rensen, Marcel den Dulk, Joost M Klaase, Steven W M Olde Damink

**Affiliations:** 1 Department of Surgery Maastricht University Medical Center+ Maastricht Netherlands; 2 Department of Surgery, School of Nutrition and Translational Research in Metabolism Maastricht University Maastricht Netherlands; 3 Department of Surgery University Medical Center Groningen Groningen Netherlands; 4 Department of Nutrition and Movement Sciences School of Nutrition and Translational Research in Metabolism Maastricht University Maastricht Netherlands; 5 Department of General, Visceral and Transplant Surgery Rheinish-Westphalian Technical University Hospital Aachen Germany

**Keywords:** preoperative care, prehabilitation, preoperative training, high-intensity interval training, pancreatic resection, cardiorespiratory fitness

## Abstract

**Background:**

Morbidity rates in pancreatic surgery are high, and frail patients with low aerobic capacity are especially at risk of complications and require prophylactic interventions. Previous studies of small patient cohorts receiving intra-abdominal surgery have shown that an exercise prehabilitation program increases aerobic capacity, leading to better treatment outcomes.

**Objective:**

In this study, we aim to assess the feasibility of a home-based exercise prehabilitation program in unfit patients scheduled for pancreatic surgery on a larger scale.

**Methods:**

In this multicenter study, adult patients scheduled for elective pancreatic surgery with a preoperative oxygen uptake (VO_2_) at the ventilatory anaerobic threshold ≤13 mL/kg/min or a VO_2_ at peak exercise ≤18 mL/kg/min will be recruited. A total of 30 patients will be included in the 4-week, home-based, partly supervised exercise prehabilitation program. The program comprises 25-minute high-intensity interval training on an advanced cycle ergometer 3 times a week. Training intensity will be based on steep ramp test performance (ie, a short-term maximal exercise test on a cycle ergometer), aiming to improve aerobic capacity. Twice a week, patients will perform functional task exercises to improve muscle function and functional mobility. A steep ramp test will be repeated weekly, and training intensity will be adjusted accordingly. Next to assessing the feasibility (participation rate, reasons for nonparticipation, adherence, dropout rate, reasons for dropout, adverse events, and patient and therapist appreciation) of this program, individual patients’ responses to prehabilitation on aerobic capacity, functional mobility, body composition, quality of life, and immune system factors will be evaluated.

**Results:**

Recruitment for this study began in January 2022 and is expected to be completed in the summer of 2023.

**Conclusions:**

Results of this study will provide important clinical and scientific knowledge on the feasibility of a partly supervised home-based exercise prehabilitation program in a vulnerable patient population. This might ease the path to implementing prehabilitation programs in unfit patients undergoing complex abdominal surgery, such as pancreatic surgery.

**Trial Registration:**

ClinicalTrials.gov NCT05496777; https://classic.clinicaltrials.gov/ct2/show/NCT05496777

**International Registered Report Identifier (IRRID):**

DERR1-10.2196/46526

## Introduction

The incidence of pancreatic malignancies is predicted to rise as the aging population grows [1]. The only curative treatment option remains pancreatic surgery, which is highly complex and known for its high postoperative morbidity [2]. Within this already vulnerable patient group, there are unfit patients with low aerobic fitness, translating into a limited reserve to withstand stress during surgery [3,4]. As a consequence, these patients are more prone to postoperative morbidity after pancreatic surgery [5,6]. To effectively identify unfit patients who are in need of preoperative intervention to reduce their perioperative risks, specific preoperative risk assessment is required. In the search for valid preoperative risk assessment diagnostics, preoperative aerobic fitness has been found to have a persistent relationship with postoperative outcomes in major elective intra-abdominal surgeries [5,7,8]. A cardiopulmonary exercise test (CPET), which determines 2 meaningful risk indicators, that is, the oxygen uptake (VO_2_) at the ventilatory anaerobic threshold (VAT) and VO_2_ at peak exercise (VO_2peak_), can be used to acquire an objective representation of a patient’s aerobic capacity [9].

Physical exercise training before surgery has been reported to effectively increase the aerobic capacity of patients scheduled for major abdominal surgery [10-17]. In the trial of Barberan-Garcia et al [18], high-risk patients scheduled for major abdominal surgery were subjected to a multimodal prehabilitation program consisting of (1) motivational interviewing, (2) a personalized program to promote daily physical activity, and (3) a supervised high-intensity endurance exercise training program. The mean duration of their program was 6 weeks, and it not only improved preoperative aerobic capacity, but also resulted in a 51% reduction in postoperative complications [18]. Regarding prehabilitation programs in pancreatic surgery, Ausania et al [14] subjected patients with a pancreatic or peripancreatic malignancy, regardless of their physical fitness, to a multimodal prehabilitation program consisting of 5 daily sessions of supervised high-intensity endurance training in the outpatient clinic. Furthermore, patients were trained to perform unsupervised home-based functional and breathing exercises, and nutritional support, as well as pancreatic exocrine insufficiency, was addressed [14]. Although the authors reported an improvement in physical fitness as determined by the 10-meter walk test, no differences in postoperative outcomes were observed [14]. However, since high-risk patients are hypothesized to benefit the most from exercise prehabilitation, adequate patient selection seems essential and greatly influences the effect of an exercise prehabilitation program on improving a patient’s aerobic capacity [11,12]. Another important factor influencing the feasibility and effect of a prehabilitation exercise program is the setting in which it takes place. Most reported prehabilitation programs have been carried out in an outpatient clinic. However, as pointed out by Ferreira et al [19], patients prefer a home-based prehabilitation program for reasons such as traveling issues or because a hospital- or community-based exercise program is regarded as too time-consuming. These perceived barriers will negatively influence a patient’s adherence to a physical exercise training program, likely resulting in a suboptimal training effect [19]. On the other hand, a possible disadvantage of unsupervised home-based exercise training could be a lack of adherence of the patient, lack of appropriate exercise intensity, and therefore lack of benefit [4]. It is therefore important to provide adequate supervision, preferably by an experienced physical therapist.

In exercise prehabilitation programs within a limited preoperative time window of 4 to 6 weeks, the most efficient training modality to increase aerobic capacity seems to be high-intensity interval training (HIIT) [17,20]. In a study by van Wijk et al [17], high-risk patients scheduled for liver or pancreatic surgery participated in a partly supervised home-based bimodal prehabilitation program consisting of HIIT and high-intensity endurance training combined with nutritional support. Their 4-week prehabilitation program was shown to be feasible and improved aerobic capacity by 17% as determined by performing a CPET pre- and postintervention.

Although several studies investigated the effect of exercise prehabilitation in major abdominal surgery, there is a large heterogeneity in patient selection and prehabilitation program setting, which notably influences its effectiveness and adequate interpretation of results. Therefore, the primary aim of this study is to determine the feasibility of a 4-week, home-based, partly supervised exercise prehabilitation program in unfit patients scheduled for pancreatic surgery. Secondary aims of the study are to evaluate the individual responses to the program on aerobic capacity, functional mobility, muscle strength, body composition, level of perceived fatigue, quality of life, and immune system factors.

## Methods

### Study Design

The described study is a multicenter feasibility study, and the study will have a pretest-posttest design. The latest version of the study protocol (version 7; May 2022) is presented in this manuscript. Protocol amendments will have to be approved by the Medical Research Ethics Committee of the Maastricht University Medical Center+ and Maastricht University (METC azM/UM).

### Eligibility Criteria

In order to be eligible to participate in this study, patients must meet all of the following criteria: (1) age 18 years or older, (2) planned for elective pancreatic surgery at Maastricht University Medical Center+ or at the University Medical Center Groningen, and (3) providing informed consent to participate. Patients are considered eligible (4) if they have a VO_2_ at the VAT ≤13 mL/kg/min or a VO_2peak_ ≤18 mL/kg/min. A patient who meets any of the following criteria will be excluded from participation in this study: (1) they require acute surgery, (2) they undergo surgery in another hospital, (3) they are not able to cycle on a cycle ergometer, (4) contraindications are identified for them to physical exercise training (eg, heart rhythm disturbances, ischemia of the heart muscle), (5) they are unable to cooperate during the testing procedures (eg, insufficient understanding of the Dutch language), and (6) no certified physical therapist is available in their living area.

### Recruitment

All patients eligible for pancreatic surgery will be identified at the multidisciplinary team meetings and evaluated by the surgeon at the outpatient clinic. During the outpatient clinic visit, patients will be given full details of the study, and after a few days, patients from the Maastricht University Medical Center+ will be contacted by telephone by a designated investigator to provide extensive information about the study. If patients are interested in participating in the study, a CPET will be performed to check whether the patient fulfills the inclusion criteria, after which an appointment will be planned to perform baseline assessments and to retrieve written informed consent. At the University Medical Center Groningen, all patients scheduled for pancreatic surgery perform a CPET as part of usual care. Based on the CPET results, patients will be asked to participate in this study and an appointment will be planned to perform the remaining baseline assessments and receive written informed consent.

### Interventions

#### Preoperative Home-Based Physical Exercise Training Program

**Figure 1:**
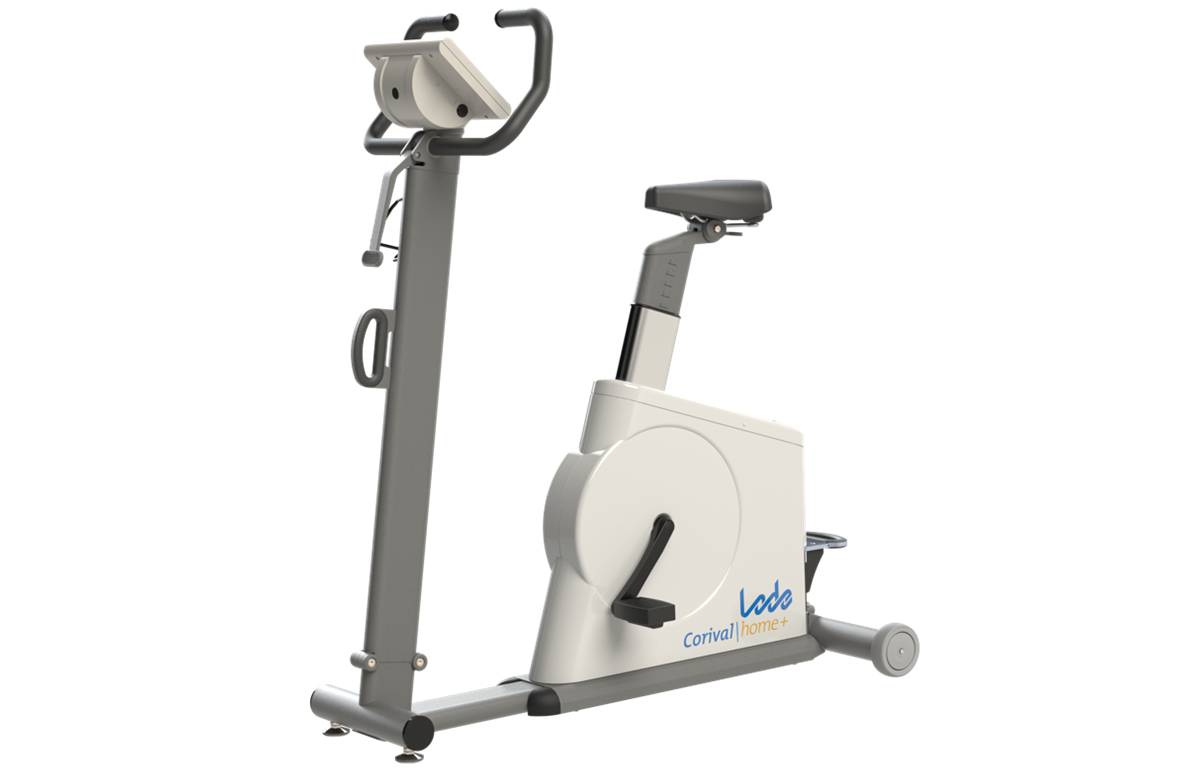
The Lode Corival Home+ cycle ergometer used in this study.

Participating patients will engage in a home-based, partly supervised exercise prehabilitation program before elective pancreatic surgery. The physical exercise training program will consist of HIIT on an advanced cycle ergometer that is placed at the patient’s home (Lode Corival Home+, Lode BV; see Figure 1), with the goal to improve the patient’s preoperative aerobic fitness. A certified community physical therapist in the living area of the patient will be asked to partly supervise the physical exercise training program. The community physical therapist will receive verbal and written instructions on the use of the cycle ergometer and the goals and content of the program.

**Figure 2:**
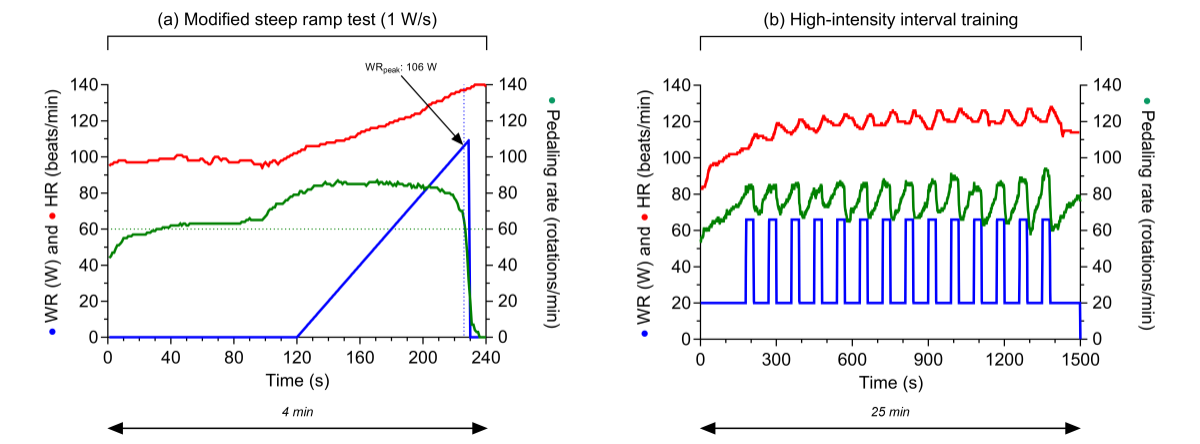
(a) Example of modified steep ramp test performance. (b) Results of a consecutive high-intensity interval training session at 60% of steep ramp test WR_peak_. HR: heart rate; WR: work rate; WR_peak_: work rate at peak exercise.

The duration of the exercise program is 4 weeks, and the training frequency will be 3 sessions per week. The supervising community physical therapist will visit the patient 3 times during the first week, and once in the weeks thereafter to monitor training progression and provide additional instructions if needed. Patients are encouraged to find a training buddy (eg, their partner or a close relative) to help and to motivate them for the unsupervised training sessions. Patients may choose the day and time point of their unsupervised training themselves, with the recommendation to leave at least one day between training sessions for recovery. This approach was chosen in order to promote adherence to the program. Each first training session of the week will be preceded by a modified steep ramp test (SRT) [21] executed under the supervision of the community physical therapist. The SRT, a short-time maximal exercise test on a cycle ergometer, has been proven to be a valid tool to estimate aerobic capacity and allows for individual optimization and progress monitoring of the HIIT program [21-23]. The training program on the cycle ergometer will be personalized weekly to each participant based on their performance during the SRT. Based on SRT performance, the consecutive training sessions comprise HIIT, consisting of a 3-minute warm-up at 20 watts, 14 high-intensity intervals of 30 seconds at 60% of the peak work rate (WR) achieved at the SRT interspersed with 14 low-intensity intervals of 60 seconds at 20 watts and a cooldown of at least 1 minute, thereby taking at least 25 minutes to execute (Table 1). Directly after each completed HIIT session of 20 minutes, the patient is asked by the program installed on the cycle ergometer to fill out the 6-20 Borg scale for rating of perceived exertion. Data about the duration, intensity (WR), heart rate, pedaling frequency, and rating of perceived exertion of each SRT and HIIT session at the cycle ergometer will be automatically recorded by the cycle ergometer at the end of each SRT and HIIT session and uploaded to a web-based platform (Figure 2). As such, community physical therapists and local investigators are able to remotely supervise and monitor training progression and adherence. Once a week, during the supervised SRT and HIIT session, the physical therapist will discuss these findings with the patient. In case of unexpected problems, the community physical therapist can consult the local investigator of the study.

**Table 1:** High-intensity interval training structure.

Exercise phase		Exercise intensity	Pedaling frequency (rotations/min)	
Warm-up (3 min)		20 W	40-80	
**Interval training (21 min)**				
	Work interval intensity (14 repetitions)	30 sec at 60% of WR_peak_^a^ of the steep ramp test	60-100	
	Rest interval intensity (14 repetitions)	60 sec at 20 W	40-60	
Cooldown (1 min^b^)		20 W	40-80	

^a^WR_peak_: work rate at peak exercise.

^b^Duration of cooldown can be extended when preferred.

#### Functional Task Exercises

Next to HIIT on the cycle ergometer, patients will be instructed to carry out functional task exercises 2 times a week on training days at which the HIIT session is not preceded by an SRT, thereby practicing activities relevant to the patient (eg, elastic band exercises, stair-climbing, brisk walking) [24]. An overview of the structure of the overall training program can be found in Table 2. The patient will be instructed to perform at least 3 repetitions of about 30 seconds of different functional task exercises with 30 seconds of rest in between. The functional task exercises will be chosen individually, consisting of activities relevant to the patient, and the intensity and complexity of exercises will be individually reviewed weekly by the physical therapist. Patients will be instructed to perform as many repetitions as intensely as possible, but at least three per functional task exercises. Next to an increasing exercise intensity or frequency, functional task exercises can also progress in difficulty in terms of making exercises more complex and by adding variation.

**Table 2:** Overall training program structure (weeks 1-4).

Training session 1 (Monday or Tuesday)		Training session 2 (Wednesday or Thursday)			Training session 3 (Friday or Saturday)	
Workout	Duration	Workout	Duration	Workout		Duration
SRT^a^	4-7 minutes	HIIT^b^	25 minutes	HIIT		25 minutes
HIIT	25 minutes	Functional task exercises	15 minutes	Functional task exercises		15 minutes

^a^SRT: steep ramp test.

^b^HIIT: high-intensity interval training.

### Usual Care

As part of usual care, all patients are screened at baseline for risk factors associated with postoperative complications and are treated accordingly. This includes screening for nutritional deficits, mental resilience, anemia, iron deficiency, hyperglycemia, and substance abuse (eg, smoking, alcohol consumption). In both participating centers, patients are screened by a trained dietitian as part of usual care. Patients at risk of malnutrition will receive additional nutritional support. When patients are in need of additional support in any of the other areas, such as psychological support or intervention for smoking cessation, this will also be implemented as a standard of care in both centers.

### Measurements

Before, during, and after the preoperative home-based physical exercise training program, several outcome measurements will be obtained (Table 3).

**Table 3:** Schedule of enrollment, intervention, and assessments.

			Preintervention	Home-based exercise prehabilitation					Postintervention		Follow-up (30 days postoperatively)
				Week 1	Week 2	Week 3	Week 4				
**Enrollment**											
		Eligibility screening	✓								
		Informed consent	✓								
**Intervention**											
		Home-based exercise prehabilitation		✓	✓	✓	✓				
**Assessments**											
		Baseline characteristics	✓								
		Feasibility	✓	✓	✓	✓	✓	✓			
	**Aerobic capacity**										
		CPET^a^	✓					✓			
		SRT^b^		✓	✓	✓	✓				
	**Muscle strength**										
		Handgrip strength	✓					✓			
	**Body composition**										
		Anthropometry	✓	✓	✓	✓	✓	✓			
		L3^c^ index	✓					✓			
	**Functional mobility**										
		30-second chair-stand test	✓	✓	✓	✓	✓	✓			
		2-minute walk test	✓					✓			
	Immunological phenotyping		✓					✓			
	**Questionnaires**										
		MFI^d^	✓					✓			
		EORTC QLQ-C30^e^	✓					✓			
		SARC-F^f^	✓					✓			
		Program appreciation^g^						✓			
	**Postoperative outcomes**										
		Surgical complications								✓	
		Length of hospital stay								✓	
		30-day readmission								✓	

^a^CPET: cardiopulmonary exercise test.

^b^SRT: steep ramp test.

^c^L3: third lumbar vertebra.

^d^MFI: Multidimensional Fatigue Index.

^e^EORTC QLQ-C30: European Organization for Research and Treatment of Cancer Quality-of-life Questionnaire Core 30.

^f^SARC-F: strength, assistance with walking, rising from a chair, climbing stairs, and falls.

^g^Patient and community physical therapist appreciation.

#### Program Feasibility

Feasibility will be determined by (1) monitoring the participation rate of this study as well as reasons for nonparticipation. Reasons for nonparticipation will be asked for during a short interview with the coordinating investigator or treating surgeon. Furthermore, feasibility will be determined by (2) evaluating adherence and fidelity of patients to the physical exercise training program as objectively recorded by the cycle ergometer, which provides insight into training session content (eg, frequency, intensity, duration, premature termination of a training session), (3) dropout rate and reasons for dropout, (4) number and severity of adverse events, and (5) patient and therapist appreciation after completing the program by filling out a short questionnaire based on a previous study [25]. If a patient drops out before the end of the training program, the reasons will be investigated through a short interview with the coordinating investigator.

#### Aerobic Capacity CPET

For all patients, a preintervention CPET will be performed by a clinical exercise physiologist (Maastricht University Medical Center) or sports physician (University Medical Center Groningen) to assess baseline aerobic capacity, which will be used to make a selection of eligible unfit patients and thereupon invite them to participate in the study (VO_2_ at the VAT ≤13 mL/kg/min or VO_2peak_ ≤18 mL/kg/min). After completion of the program, a postintervention CPET will be performed. The outcome thereof will be used to assess the effectiveness of the home-based physical exercise training program to improve aerobic capacity.

The CPET will be executed under controlled conditions using a calibrated electronically braked cycle ergometer in an upright position; the Lode Corival CPET (Lode BV) will be used at Maastricht University Medical Center and the Lode Excalibur Sport (Lode BV) will be used at the University Medical Center Groningen. Patients will be fitted with a 12-lead electrocardiogram to rule out strain-related cardiac ischemia, arrhythmias, and other contraindications for intense physical exercise training during the consecutive exercise program. During the test, patients will breathe through a face mask connected to a metabolic cart; the Vyntus CPX (Vyaire Medical) will be used at the Maastricht University Medical Center and the Quark CPET (Cosmed) will be used at the University Medical Center Groningen for measurements of breath-by-breath VO_2_, minute ventilation, and carbon dioxide production throughout the CPET. Finally, heart rate and blood pressure will be monitored, and peripheral oxygen saturation will be measured at the index finger. After 2 minutes of rest measurements, the patient is asked to start cycling. The first 2 minutes consist of unloaded cycling. Thereafter, the WR will be linearly incremented with a 5, 10, 15, or 20 W/min ramp protocol (depending on the patient’s self-reported physical fitness) to ensure a test duration between 8 and 12 minutes. Patients will be instructed to maintain a pedaling frequency of around 80 revolutions/min. The test effort will be considered maximal when the patient shows objective signs (ie, heart rate at peak exercise >95% of predicted or a respiratory exchange ratio at peak exercise >1.10) or subjective signs (eg, unsteady biking, sweating, clear unwillingness to continue exercising despite strong encouragement) of maximal effort. CPET interpretation will be performed according to international guidelines by an experienced sports physician or medical physiologist [26]. The last 30 seconds before termination of the test will be used to calculate absolute values at peak exercise. Peak heart rate is the highest heart rate achieved throughout the test. The VAT is described as the point at which the partial end-tidal oxygen tension and the ventilatory equivalent for oxygen reached a minimum and thereafter began to rise in a consistent manner, corresponding with an unchanged ventilatory equivalent for partial end-tidal carbon dioxide tension and carbon dioxide [26]. If this method provides unreliable results, the V-slope method will be applied instead [27].

#### Aerobic Capacity—Modified SRT

During the physical exercise training program at the patient’s home, the physical therapist will assess and monitor aerobic fitness by performing a modified SRT [21] on a weekly basis on the advanced cycle ergometer (Lode Corival Home+; Lode BV) in an upright position. Training intensity will be adjusted based on SRT performance, which is expected to maintain a sufficient training stimulus. A heart rate belt will be fitted around the patient’s chest (Xand Cycling) during the test. The SRT starts with 2 minutes of unloaded cycling. Next, the WR will be increased in a ramp-like manner by a constant increment of 10 W/10 s (1 W/s). The total test duration is estimated to be 4 to 7 minutes. The patient is instructed to keep the pedaling frequency constant between 60 and 80 revolutions/minute. The main outcome of the SRT is the work rate at peak exercise (WR_peak_), defined as the point at which there is a sustained drop in pedaling frequency from 60 revolutions/min despite strong verbal encouragement. After attaining their WR_peak_, patients will be asked their level of perceived exertion by filling out the 6-20 Borg scale for rating of perceived exertion. They will conclude the SRT with a 2-minute cooldown phase consisting of unloaded cycling at a pedaling frequency of about 40 revolutions/min.

#### Muscle Strength

To assess the effect of the exercise prehabilitation program on muscle strength, handgrip strength (in kilograms) will be measured at baseline and postintervention using the Jamar dynamometer (Sammons Preston; Rolyon) [28]. Muscle strength, as measured by handgrip dynamometry, is considered to be an indicator of frailty and an independent predictor of complications after surgery [29,30]. Patients will be asked to sit in an upright position with the elbows stretched in a straight line downwards. Patients will be asked to squeeze the handle as forcefully as possible for about 2 seconds, starting with the dominant hand. A total of 3 grip measurements per hand will be performed, with a 15- to 20-second pause between each measurement.

#### Body Composition

The patient’s body height (determined to the nearest 0.5 cm) and body mass (determined to the nearest 0.1 kg) will be measured with a metric measuring tape with a wall stop and an electronic scale (Seca 803; Seca), respectively, at baseline and postintervention. During the prehabilitation program, the community physical therapist will measure the patient’s body mass weekly.

Myosteatosis (ie, skeletal muscle fat infiltration) is a biomarker for cancer cachexia (ie, a multifactorial wasting syndrome leading to loss of skeletal muscle mass with or without loss of fat mass, subsequently inducing progressive functional impairment) and sarcopenia (ie, loss of skeletal muscle tissue). Myosteatosis alone, as well as cancer cachexia and sarcopenia, are associated with increased morbidity and mortality [31,32]. Meanwhile, it has been suggested that physical exercise training improves muscle quality [33]. Therefore, we will evaluate skeletal muscle quantity and quality at baseline and after prehabilitation by analyzing a single slice of a computerized tomography (CT) scan at the midlevel of the third lumbar vertebra (L3), which is where both transverse processes are visible. Body composition analysis will be performed using an automated segmentation system (Mosamatic) developed and validated at Maastricht University [34,35]. Visceral adipose tissue, subcutaneous adipose tissue, and skeletal muscle tissue will be normalized for the patient’s body height to calculate the L3 index in cm^2^/m^2^. Radiation attenuation will be determined by calculating the average Hounsfield unit value of the areas. Low skeletal muscle radiation attenuation is associated with increased myosteatosis [36]. For this study, the CT scans that are part of the standardized diagnostic work-up of patients will be analyzed. Since CT imaging prior to oncologic surgery should not be older than 6 weeks, preoperative CT scans are performed on a regular basis in patients.

#### Functional Mobility

Functional mobility will be assessed using the 2-minute walk test and 30-second chair-stand test at baseline and postintervention at the hospital, as well as weekly at the patient’s home. The 30-second chair-stand test evaluates a combination of lower leg muscle strength, balance, and functional mobility by testing the number of repetitions standing up from a sitting position within 30 seconds [37]. The 2-minute walk test is a feasible method to record the distance (in meters) that is walked over a standardized trail in 2 minutes [38].

#### Immunological Phenotyping

As the immune system is highly responsive to physical exercise, it has been suggested that structural physical exercise training can improve overall health and may prolong survival in patients with cancer [39]. To exploratively quantify the effect of the preoperative physical exercise training program on the immune system response, blood samples will be taken at baseline and postintervention and analyzed for levels of C-reactive protein, tumor necrosis factor-α, as well as interleukin-6, interleukin-8, and interleukin-10 by multiplex analysis. Moreover, a separate blood sample for each patient will be drawn for storage at –80 °C for future analysis. Blood samples will be stored in the biobank of the participating hospital, for a maximum of 10 years. Future analysis has to be in line with the aims of our ongoing research; if not, informed consent must be retrieved.

#### Questionnaires

Prior to the start of the physical exercise training program, patients are asked to fill out a perceived fatigue, quality of life, and sarcopenia questionnaire. The level of perceived fatigue is assessed using the Multidimensional Fatigue Index [40], quality of life will be evaluated with the European Organization for Research and Treatment of Cancer Quality-of-life Questionnaire Core 30 (EORTC QLQ-C30) [41], and finally, the strength, assistance with walking, rising from a chair, climbing stairs, and falls (SARC-F) questionnaire [42] is used to screen for sarcopenia in patients. To check for potential improvements in perceived fatigue, quality of life, and sarcopenia, these questionnaires will be repeated after completion of the exercise prehabilitation program.

### Study Outcomes

#### Primary Outcome

The primary outcome of this study is to assess the feasibility of a 4-week, home-based, partly supervised exercise prehabilitation program in unfit patients planned for elective pancreatic surgery. Feasibility is assessed via the participation rate, and by recording reasons for nonparticipation, adherence or compliance and fidelity to the program, dropout rate and reasons for dropout, adverse events during the program, and patient and therapist appreciation.

#### Secondary Outcomes

Secondary outcomes are to evaluate the patients’ responses to exercise prehabilitation on aerobic capacity, functional mobility, muscle strength, body composition, perceived fatigue, quality of life, immune system factors, number of overall postoperative complications within 30 days after surgery, and readmissions within 30 days after discharge. Complications will be graded using the Clavien-Dindo classification and divided into surgical and nonsurgical complications [43,44]. The comprehensive complication index, a continuous scale that also takes into account the number of complications per patient, will be used to summarize complications for each patient [45].

#### Other Study Outcomes

Data on patient characteristics (eg, age, sex, nutritional status, Charlson Comorbidity Index, American Society of Anesthesiologists score, smoking, location, and type of the tumor) neoadjuvant therapy, and surgical procedure will also be collected for explorative purposes.

#### Safety

During the preoperative physical exercise training program and 30 days after completion of the program, all adverse events observed by the investigator or his staff or reported spontaneously by the participating patients will be recorded.

### Data Analysis

#### Sample Size Calculation

This study will specifically evaluate the feasibility of a home-based, partly supervised prehabilitation program in unfit patients undergoing elective pancreatic surgery. Thus, the study has a clear explorative character and will analyze individual responses to the program. Based on the number of patients undergoing elective pancreatic surgery annually and the relatively short study period (<1 year), 15 patients who are willing to participate in the study will be included per hospital, resulting in a total of 30 patients. Results of this exploratory study will provide information on how to proceed in subsequent effect studies. Furthermore, results can be used to optimize the home-based prehabilitation program in the future.

#### Procedures for Data Checking and Entering

All data will be handled confidentially according to the General Data Protection Regulation (GDPR). Signed informed consent forms and other traceable data will be stored on a local server secured with a password. Coded data will be collected in an electronic case report form created in Castor (Castor), a program aimed at clinical trial data recording and monitoring. Only the principal and coordinating investigator of each center will have access to uncoded data. Variables will be checked for the number of missing, improbable, or impossible values prior to statistical analysis. In case of impossible or improbable values, the patient’s data file will be reviewed.

#### Statistical Analysis

The R software package (R Foundation for Statistical Computing) will be used for statistical analysis. Collected data on patient characteristics will be presented in tables. Continuous variables will be displayed as mean (SD), while categorical variables will be presented as number (n) and percentage (%). Descriptive statistics will be used to answer the primary study parameter of feasibility. As previously described, feasibility will be presented in tables and based on participation rate (n, %), reasons for nonparticipation (n, %, text), adherence to the program (%), dropout rates (%), reasons for dropout (n, %, text), adverse events (n, %, text), patient motivation (scale 0-10), and appreciation (text). The secondary aim is to evaluate the patients’ responses over time in terms of aerobic capacity, muscle function, functional mobility, body composition, perceived fatigue, quality of life, and immune system function. Individual response profiles will be graphically depicted and presented in tables. A repeated measurements analysis will be used to analyze changes over time in continuous variables. *P* values <.05 will be considered statistically significant.

#### Dissemination Policy

Research results will be disclosed and submitted to a peer-reviewed scientific journal, in case of positive as well as negative results. The investigators will prepare the manuscript together. Coauthorship is preserved for all participating investigators and those who at the discretion of the principal investigator constructively contributed to the study. Disputes on the interpretation of results will not lead to an unnecessary delay in publication.

### Ethics Approval

This study was approved by the ethical committee METC azM/UM in the Netherlands (METC20-090, NL75340.068.20, September 2021) and is registered in the ClinicalTrials.gov register (NCT05496777). Informed consent will be obtained from all participating subjects. All methods are carried out according to relevant guidelines and regulations.

## Results

Recruitment started in January 2022 and will run until patient inclusion is complete (expected in the summer of 2023) at the Maastricht University Medical Center+ (Netherlands) and the University Medical Center Groningen (Netherlands). After data analysis, results on all relevant study outcomes will be published.

## Discussion

Results of this study will provide important clinical knowledge on implementing a supervised (partly direct supervision, partly remote supervision) home-based exercise prehabilitation program for unfit patients scheduled for pancreatic surgery. Any potential barriers that hamper the effective execution of a physical exercise training program will be identified by thoroughly analyzing feasibility outcomes. The resulting scientific knowledge will fill in gaps in this literature and will provide the ability to improve preoperative optimization of unfit patients. Proof of feasibility might ease the path to implementing exercise prehabilitation programs in patients undergoing complex abdominal surgery, such as pancreatic surgery. Ultimately, this may lead to better treatment outcomes in vulnerable patient populations. Apart from future patients being scheduled for pancreatic surgery, we expect participating patients to benefit directly from the physical exercise training program due to an expected increase in preoperative aerobic capacity and subsequently have better treatment outcomes.

Several studies indicated that a VO_2_ at the VAT <11 mL/kg/min or a VO_2peak_ <18 mL/kg/min, as determined during a CPET, are accurate cut-off points to identify patients that have a higher risk for postoperative morbidity and mortality [5,8]. Nevertheless, preoperative physical exercise training might also be beneficial for patients with mediocre aerobic capacity undergoing pancreatic surgery [46]. In the Netherlands, the mean waiting time for surgery is 4 to 6 weeks, meaning there is sufficient time for patients to participate in physical exercise training programs. Therefore, we have chosen to include all patients with a VO_2_ at the VAT ≤13 mL/kg/min or a VO_2peak_ ≤18 mL/kg/min in their waiting period. Previous studies have clearly demonstrated the beneficial effect of exercise prehabilitation on aerobic capacity in patients scheduled for intra-abdominal surgery [13-16,18]. In high-risk patients scheduled for major abdominal surgery, Barberan-Garcia et al [18] demonstrated a 35% increase in aerobic endurance time and a 51% reduction of postoperative complications after a 6-week course of preoperative high-intensity endurance training. More recently, Berkel et al [16] demonstrated a 10% increase in aerobic capacity (VO_2_ at the VAT and VO_2peak_) and an almost 50% decrease in the incidence of postoperative complications in high-risk patients scheduled for colorectal surgery undergoing a 3-week community-based and personalized preoperative HIIT program. Despite evidence suggesting that exercise prehabilitation is effective, there is a large heterogeneity in design and setting among reported prehabilitation programs. Although most of the reported prehabilitation programs were carried out under supervision in an outpatient clinic, patients clearly prefer a home-based prehabilitation program [19]. Nevertheless, home-based prehabilitation programs should be supervised, since a possible disadvantage of unsupervised home-based exercise training could be a lack of adherence of the patient or lack of appropriate exercise intensity and therefore lack of benefit [4]. A supervised and personalized exercise program in a home-based setting might enhance the participation rate, adherence, and motivation of (high-risk) patients, and has been shown to be the preferred method for a prehabilitation program [4,17,47 ]. This was illustrated by van Wijk et al [17], who recently reported an 83% adherence rate in their partly supervised home-based training program, which led to an increase of respectively 17.8% and 17.2% in VO_2_ at the VAT and VO_2peak_ in unfit (VO_2_ at the VAT <11 mL/kg/min) patients undergoing liver or pancreatic resection.

Strengths of this study are its clear study design consisting of a supervised home-based prehabilitation program, the ability to thoroughly assess feasibility in a vulnerable patient population, the use of validated measurement instruments, and the personalized training program based on an individual patient’s performance at the weekly executed SRT. This study aims to set the standard for home-based exercise prehabilitation programs in pancreatic surgery. Nevertheless, this study will have some limitations. First of all, the physical exercise training program is only partly supervised by a physical therapist. This may lead to a lower patient adherence to the exercise program and subsequently suboptimal training results. However, the advanced cycle ergometer used in this study automatically captures data on training frequency, intensity, and duration. This enables the physical therapist to monitor training progression and compliance in real-time and gives the opportunity to intervene if necessary. Furthermore, as mentioned before, van Wijk et al [17] have reported an acceptable 83% adherence in their partly supervised exercise program. In addition, given that the primary outcome of interest is the feasibility of the training program, noncompletion of training adds valuable information to the data set. Another limitation might be that our study focuses on exercise prehabilitation while most unfit patients might benefit from a multimodal approach, including screening and subsequent treatment of common risk factors associated with postoperative complications. This screening is part of the usual care in both centers. If necessary, nutritional support, treatment of modifiable risk factors (eg, dyslipidemia, smoking, alcohol consumption), and mental support will be implemented within the patient’s individual prehabilitation program.

While hospital- and community-based exercise prehabilitation programs encounter significant feasibility issues when enrolling vulnerable unfit patients, this study will offer unfit patients a unique personalized exercise prehabilitation program with adequate supervision in the setting of their own residence. Hence, it is expected that this study will achieve high adherence rates and subsequently high efficacy in improving patients’ preoperative aerobic capacity.

## Abbreviations

CPET: cardiopulmonary exercise test
CT: computerized tomography
EIT: European Institute of Innovation and Technology
EORTC QLQ-C30: European Organization for Research and Treatment of Cancer Quality-of-life Questionnaire Core 30
GDPR: General Data Protection Regulation
HIIT: high-intensity interval training
L3: third lumbar vertebra
MFI: Multidimensional Fatigue Index
SARC-F: strength, assistance with walking, rising from a chair, climbing stairs, and falls
SRT: steep ramp test
VAT: ventilatory anaerobic threshold
VO2: oxygen uptake
VO2peak: oxygen uptake at peak exercise
WR: work rate
WR_peak_: work rate at peak exercise

## Data Availability

Data sharing is not applicable to this article as no data sets have yet been generated or analyzed.
